# 4-(3-Methyl­anilino)-*N*-[*N*-(1-methyl­ethyl)carbamo­yl]pyridinium-3-sulfon­amidate (torasemide) methanol 0.25-solvate 0.25-hydrate

**DOI:** 10.1107/S160053680901160X

**Published:** 2009-04-08

**Authors:** Gianluca Bartolucci, Bruno Bruni, Silvia A. Coran, Massimo Di Vaira

**Affiliations:** a Dipartimento di Scienze Farmaceutiche, Universitá di Firenze, Via U. Schiff 6, I-50019 Sesto Fiorentino, Firenze, Italy; b Dipartimento di Chimica, Universitá di Firenze, Via della Lastruccia 3, I-50019 Sesto Fiorentino, Firenze, Italy

## Abstract

The title compound, C_16_H_20_N_4_O_3_S·0.25CH_4_O·0.25H_2_O, is a hydrate/methanol solvate of torasemide, a diuretic drug used in the treatment of hypertension. The asymmetric unit contains two torasemide mol­ecules and half-occupied methanol and water mol­ecules. It is isomorphous with the previously reported nonsolvated T–II form of torasemide. The water mol­ecules contribute to the stability of the structure by participating in an extensive system of O—H⋯O hydrogen bonds; N—H⋯N and N—H⋯O hydrogen bonds are also present. Both asymmetric mol­ecules of torasemide form inversion dimers in the crystal.

## Related literature

For background on the medicinal properties and polymorphism of torasemide, see: Uchida *et al.* (1991 ▶); Broekhuysen *et al.* (1986 ▶); Ghys *et al.* (1985 ▶); Ishido & Senzaki (2008 ▶); Cosin & Diez (2002 ▶); Murray *et al.* (2001 ▶); Dupont *et al.* (1978 ▶); Danilovski *et al.* (2001 ▶).
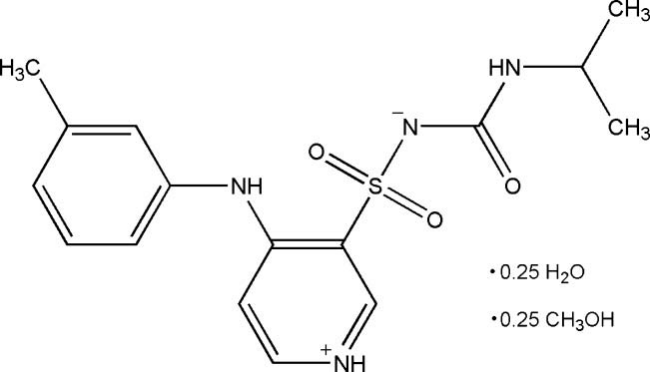


## Experimental

### 

#### Crystal data

C_16_H_20_N_4_O_3_S·0.25CH_4_O·0.25H_2_O
*M* *_r_* = 360.94
Monoclinic, 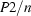

*a* = 16.8477 (2) Å
*b* = 11.5951 (1) Å
*c* = 20.3256 (2) Å
β = 108.646 (1)°
*V* = 3762.21 (7) Å^3^
*Z* = 8
Cu *K*α radiation
μ = 1.74 mm^−1^
*T* = 200 K
0.45 × 0.38 × 0.12 mm

#### Data collection

Oxford Diffraction Xcalibur PX Ultra CCD diffractometer
Absorption correction: multi-scan (*ABSPACK*; Oxford Diffraction, 2006 ▶) *T*_min_ = 0.502, *T*_max_ = 1.000 (expected range = 0.407–0.811)
50677 measured reflections
7402 independent reflections
6976 reflections with *I* > 2σ(*I*)
*R*_int_ = 0.031

#### Refinement

*R*[*F* ^2^ > 2σ(*F* ^2^)] = 0.052
*wR*(*F* ^2^) = 0.156
*S* = 1.04
7402 reflections
496 parameters
6 restraints
H atoms treated by a mixture of independent and constrained refinement
Δρ_max_ = 0.95 e Å^−3^
Δρ_min_ = −0.57 e Å^−3^

### 

Data collection: *CrysAlisPro CCD* (Oxford Diffraction, 2006 ▶); cell refinement: *CrysAlisPro CCD*; data reduction: *CrysAlisPro RED* (Oxford Diffraction, 2006 ▶); program(s) used to solve structure: *SIR97* (Altomare *et al.*, 1999 ▶); program(s) used to refine structure: *SHELXL97* (Sheldrick, 2008 ▶); molecular graphics: *ORTEP-3* (Farrugia, 1997 ▶) and *PLATON* (Spek, 2009 ▶); software used to prepare material for publication: *SHELXL97* (Sheldrick, 2008 ▶), *WinGX* (Farrugia, 1999 ▶) and *PARST* (Nardelli, 1995 ▶).

## Supplementary Material

Crystal structure: contains datablocks global, I. DOI: 10.1107/S160053680901160X/hb2934sup1.cif

Structure factors: contains datablocks I. DOI: 10.1107/S160053680901160X/hb2934Isup2.hkl

Additional supplementary materials:  crystallographic information; 3D view; checkCIF report

## Figures and Tables

**Table 1:** Hydrogen-bond geometry (Å, °)

*D*—H⋯*A*	*D*—H	H⋯*A*	*D*⋯*A*	*D*—H⋯*A*
N1—H1*N*⋯N3	0.83 (3)	2.37 (3)	2.986 (2)	131 (2)
N1—H1*N*⋯N3^i^	0.83 (3)	2.38 (3)	3.060 (2)	139 (2)
N2—H2*N*⋯O5^ii^	0.84 (3)	2.14 (3)	2.847 (2)	142 (3)
N2—H2*N*⋯O6^ii^	0.84 (3)	2.21 (3)	2.822 (2)	130 (2)
N4—H4*N*⋯O1^i^	0.90 (3)	2.02 (3)	2.919 (2)	174 (3)
N5—H5*N*⋯N7	0.86 (3)	2.21 (3)	2.910 (2)	138 (2)
N5—H5*N*⋯N7^iii^	0.86 (3)	2.47 (3)	3.098 (2)	130 (2)
N6—H6*N*⋯O2^iv^	0.92 (3)	2.53 (3)	3.036 (2)	115 (2)
N6—H6*N*⋯O3^iv^	0.92 (3)	1.79 (3)	2.667 (2)	158 (2)
N8—H8*N*⋯O4^iii^	0.85 (3)	2.21 (3)	3.034 (3)	164 (3)
O7—H7*O*⋯O8	0.923 (8)	1.70 (5)	2.480 (8)	140 (7)
O7—H7*O*⋯O8^v^	0.923 (8)	2.50 (10)	2.995 (9)	114 (8)
O8—H82*O*⋯O6	0.75 (5)	2.00 (6)	2.735 (5)	166 (11)
O8—H81*O*⋯O6^v^	0.75 (5)	2.02 (6)	2.721 (5)	158 (12)

Symmetry codes: (i) 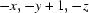; (ii) 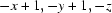; (iii) 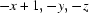; (iv) 
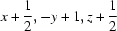
; (v) 
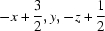
.

**Fig. 1.**
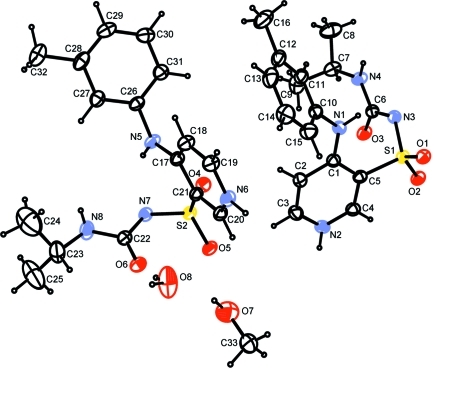
A view of the content of the asymmetric unit of (I), where the sites of the solvate water and methanol molecules have 0.50 occupancy. Displacement ellipsoids are drawn at the 50% probability level.

**Fig. 2.**
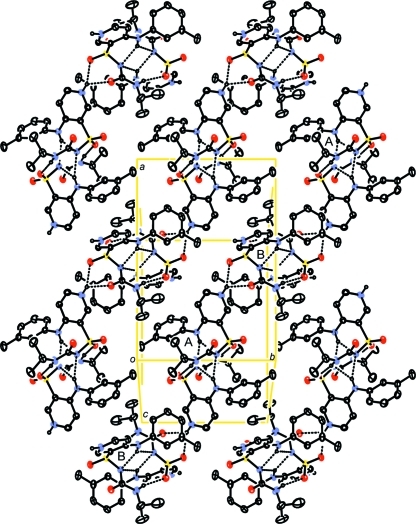
A view of the crystal packing in the structure of (I), in proximity of the ab face. Hydrogen bonds are denoted by dashed lines. Only hydrogen atoms involved in the formation of hydrogen bonds are shown. Chains of hydrogen–bonded molecules, parallel to the [110] direction, are formed by centrosymmetric dimers of the two types of symmetry–independent molecules (denoted by the A and B letters, respectively). The solvent molecules, not shown in this drawing, lie between this layer and the parallel one at c = 1/2, where the chains of hydrogen–bonded molecules exhibit the alternative [1–10] orientation.

**Fig. 3.**
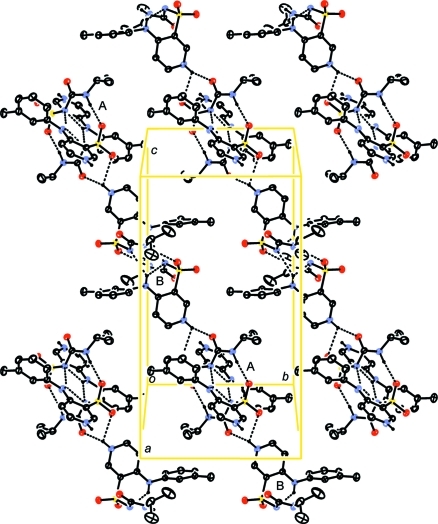
The arrangement of chains, parallel to the [01–1] direction, of hydrogen–bonded molecules in proximity of the bc face. A similar arrangement, however with the alternative [011] chain orientation, exists on the parallel flanking planes at a/2 distance from the one shown.

**Fig. 4.**
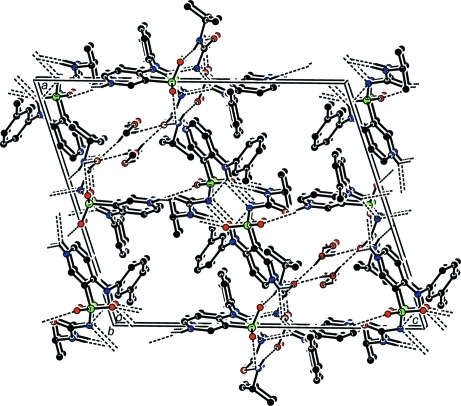
A view of the packing in the structure of (I) showing channels hosting the solvent molecules. Dimers of A or B molecules aligned along b, according to this view, are not connected by hydrogen bonds.

## supplementary crystallographic information

### Comment

Torasemide has been developed as a longlasting loop diuretic that combines the effects of both furosemide and spironolactone (Uchida *et al.*, 1991; Broekhuysen *et al.*, 1986; Ghys *et al.*, 1985). It is used in the treatment of hypertension and of edema associated with congestive heart failure (Ishido & Senzaki, 2008). Torasemide has been reported to be more effective than furosemide in chronic heart failure, with a lower mortality (Cosin & Diez, 2002; Murray *et al.*, 2001).

Three polymorphic forms have been reported up to now for torasemide, denoted T–I, T–II (Dupont *et al.*, 1978) and T–N (Danilovski *et al.*, 2001). We have obtained crystals of a solvate (I) which, besides being isomorphous with form T–II (with the *a* and *c* unit cell axes interchanged), contains water and methanol molecules interspersed in the lattice. The solvate molecules are disorderly arranged in proximity of a twofold rotation axis and one molecule of each type resides, with 0.50 occupancy, in the asymmetric unit of the monoclinic unit cell which comprises, in addition, two independent torasemide molecules (Fig. 1), as found for form T–II. Although data collection for I was carried out at a temperature of 200 K, only a 0.8% decrease in the cell volume was found with respect to the room–temperature value of T–II; this might be ascribed in part to the presence of the additional solvate molecules in the lattice of I. The 200 K data collection temperature represented a compromise between conditions of high thermal motion at room temperature, particularly affecting the terminal methyl groups of the chain, and crystal deterioration at lower temperatures. The conformations of the two independent molecules in the structure of I (respectively formed by carbon atoms C1 to C16 and C17 to C32, hereafter referred to as molecules A and B, in the above order, for consistency with previous notation) are similar to those of the A and B molecules of the T–II form, with an 8.4° largest difference in the value of the C22—N8—C23—C24 torsion angle (this corresponds to the C6—N4—C7—C9 chain of molecule B of the T–II form, according to Dupont's notation for the non–solvated form). The present values of the dihedral angles between the best planes through the phenyl and pyridyl rings in the two molecules, of 61.18 (7)° (A) and 78.71 (7)° (B), match those reported (61.2° and 79.1°) for the corresponding molecules of form T—II.

It should be noted that the present set of data, contrary to the older one for T–II, has allowed to assign unambiguously and refine the positions of all hydrogen atoms attached to the N atoms in the two molecules. In particular, the N—H amine bond formed by N1 in molecule A and by N5 in B, is almost parallel, in each case, to the plane of the pyridyl ring, yielding a substantially planar immediate environment of the nitrogen atom, whereas such bond is considerably displaced from the plane of the phenyl ring. This may rationalize the large difference, *ca* 0.09 Å, between the lengths of the two N—C bonds formed by N1 and, respectively, N5 in the two molecules. Indeed, as a consequence of the above N—H bond orientations, the nitrogen lone pair may favourably interact with antibonding orbitals of the pyridyl ring, but not with those of the phenyl ring, yielding shorter N1—C1 and N5—C17 bonds compared to the N1—C10 and N5—C26 ones (with the additional consequence that the two contiguous C—C bonds in each pyridyl ring are the longest of all bonds in the present rings).

The crystal structure is stabilized by an extensive system of hydrogen bonds (Table 1), several of these in bifurcated mode. There are centrosymmetric dimers of molecule A, internally connnected by the N1···N3 (N1···N3 = 2.986 (2) Å, N1—H1N···N3 = 131 (2)°), N1···N3^i^ (N1···N3^i^ = 3.060 (2) Å, N1—H1N···N3^i^ = 139 (2)°; symmetry code (i): - *x*, 1 - *y*, - *z*) and N4···O1^i^ (N4···O1^i^ = 2.919 (2) Å, N4—H4N···O1^i^ = 174 (3)°) hydrogen bonds and centrosymmetric dimers of molecule B, linked by the N5···N7 (N5···N7 = 2.910 (2) Å, N5—H5N···N7 = 138 (2)°), N5···N7^iii^ (N5···N7^iii^ = 3.098 (2) Å, N5—H5N···N7^iii^ = 130 (2)°; symmetry code (iii): 1 - *x*, - *y*, - *z*) and N8···O4^iii^ (N8···O4^iii^ = 3.034 (3) Å, N8—H8N···O4^iii^ = 164 (3)° hydrogen bonds. Dimers of the above two types, connected through the N2···O5^ii^ (N2···O5^ii^ = 2.847 (2) Å, N2—H2N···O5^ii^ = 142 (3)°; symmetry code (ii): 1 - *x*, 1 - *y*, - *z*) and N2···O6^ii^ (N2···O6^ii^ = 2.822 (2) Å, N2—H2N···O6^ii^ = 130 (2)°) hydrogen bonds, form chains in an AABBAA fashion, which are stacked, with no intervening hydrogen bonds among them, on planes parallel to the *ab* cell face (Fig. 2). On contiguous planes of this set, spaced by *c*/2 intervals, the chains are alternatively parallel to the [110] and [1–10] directions. Furthermore, a three–dimensional network arises since dimeric groups from the above adjacent planes are connected through the N6···O2^iv^ (N6···O2^iv^ = 3.036 (2) Å, N6—H6N···O2^iv^ = 115 (2)°; symmetry code (iv): 1/2 + *x*, 1 - *y*, 1/2 + *z*) and N6···O3^iv^ (N6···O3^iv^ = 2.667 (2) Å, N6—H6N···O3^iv^ = 158 (2)°) linkages. Also the latter system of connections gives rise to planar arrays of AABBAA chains, these being parallel to the *bc* cell face (Fig. 3). Also in this case, there are alternative [01–1] and [011] chain orientations on adjacent planes, at *a*/2 intervals. It is remarkable that a similar arrangement (with one exception, see the accompanying paper) is found for the structure of the T–N polymorph, in spite of quite different cell settings. The (disordered) water molecule bridges between the chains (Fig. 4), through the O8···O6 (O8···O6 = 2.735 (5) Å, O8—H82O···O6 = 166 (11)°) and O8···O6^v^ (O8···O6^v^ = 2.721 (5) Å, O8—H81O···O6^v^ = 158 (12)°; symmetry code (v): 3/2 - *x*, *y*, 1/2 - *z*) hydrogen bonds and each water fraction accepts one such bond, O7···O8 (O7···O8 = 2.480 (8) Å, O7—H7O···O8 = 140 (7)°), from the closest fraction of the methanol molecule.

### Experimental

Samples of torasemide were kindly provided by SIMS (SIMS srl, Reggello Firenze, Italy). Crystals of (I), in the form of colourless prisms suitable for X-ray diffraction analysis, were obtained by slow evaporation from 2:1 methanol:butanol solutions. The presence of a small amount of water molecules in the structure may be rationalized considering that the operations were not performed in completely anhydrous conditions.

### Refinement

H atoms bound to carbon atoms were in geometrically generated positions, riding. The coordinates of those bound to the N atoms of the torasemide molecules were refined freely, whereas those of H atoms of the solvent molecules were refined with geometric restraints. The constraint *U(H)* = 1.2*U*_eq_(C,*N*) on hydrogen temperature factors was applied [*U(H)* = 1.5*U*_eq_(C) for the H atoms of methyl groups and solvate molecules]. The N—H bond distances formed by refined hydrogen atoms were in the range 0.835 – 0.917 Å, the H_2_O O—H bonds refined to 0.75 (5) Å and the methanol O—H to 0.923 (8) Å.

### Crystal data

**Table:**

C_16_H_20_N_4_O_3_S·0.25CH_4_O·0.25H_2_O	*F*(000) = 1528
*M**_r_* = 360.94	*D*_x_ = 1.274 Mg m^−^^3^
Monoclinic, *P*2/*n*	Cu *K*α radiation, λ = 1.54180 Å
Hall symbol: -P 2yac	Cell parameters from 37208 reflections
*a* = 16.8477 (2) Å	θ = 4.1–72.4°
*b* = 11.5951 (1) Å	µ = 1.74 mm^−^^1^
*c* = 20.3256 (2) Å	*T* = 200 K
β = 108.646 (1)°	Prism, colorless
*V* = 3762.21 (7) Å^3^	0.45 × 0.38 × 0.12 mm
*Z* = 8	

### Data collection

**Table:**

Oxford Diffraction Xcalibur PX Ultra CCD diffractometer	7402 independent reflections
Radiation source: fine-focus sealed tube	6976 reflections with *I* > 2σ(*I*)
Oxford Diffraction Enhance ULTRA assembly	*R*_int_ = 0.031
Detector resolution: 8.1241 pixels mm^-1^	θ_max_ = 72.9°, θ_min_ = 4.1°
ω scans	*h* = −17→20
Absorption correction: multi-scan (ABSPACK; Oxford Diffraction, 2006)	*k* = −13→13
*T*_min_ = 0.502, *T*_max_ = 1.000	*l* = −25→25
50677 measured reflections	

### Refinement

**Table:**

Refinement on *F*^2^	Secondary atom site location: difference Fourier map
Least-squares matrix: full	Hydrogen site location: inferred from neighbouring sites
*R*[*F*^2^ > 2σ(*F*^2^)] = 0.052	H atoms treated by a mixture of independent and constrained refinement
*wR*(*F*^2^) = 0.156	*w* = 1/[σ^2^(*F*_o_^2^) + (0.0978*P*)^2^ + 2.03*P*] where *P* = (*F*_o_^2^ + 2*F*_c_^2^)/3
*S* = 1.04	(Δ/σ)_max_ = 0.001
7402 reflections	Δρ_max_ = 0.95 e Å^−^^3^
496 parameters	Δρ_min_ = −0.57 e Å^−^^3^
6 restraints	Extinction correction: *SHELXL97* (Sheldrick, 2008), Fc^*^=kFc[1+0.001xFc^2^λ^3^/sin(2θ)]^-1/4^
Primary atom site location: structure-invariant direct methods	Extinction coefficient: 0.00135 (17)

### Special details

**Table:**

Geometry. All e.s.d.'s (except the e.s.d. in the dihedral angle between two l.s. planes) are estimated using the full covariance matrix. The cell e.s.d.'s are taken into account individually in the estimation of e.s.d.'s in distances, angles and torsion angles; correlations between e.s.d.'s in cell parameters are only used when they are defined by crystal symmetry. An approximate (isotropic) treatment of cell e.s.d.'s is used for estimating e.s.d.'s involving l.s. planes.
Refinement. Refinement of *F*^2^ against ALL reflections. The weighted *R*-factor *wR* and goodness of fit *S* are based on *F*^2^, conventional *R*-factors *R* are based on *F*, with *F* set to zero for negative *F*^2^. The threshold expression of *F*^2^ > σ(*F*^2^) is used only for calculating *R*-factors(gt) *etc*. and is not relevant to the choice of reflections for refinement. *R*-factors based on *F*^2^ are statistically about twice as large as those based on *F*, and *R*- factors based on ALL data will be even larger.

### Fractional atomic coordinates and isotropic or equivalent isotropic displacement parameters (Å^2^)

**Table:**

	*x*	*y*	*z*	*U*_iso_*/*U*_eq_	Occ. (<1)
C1	0.19533 (12)	0.48508 (17)	−0.00966 (10)	0.0373 (4)	
C2	0.27341 (13)	0.43982 (19)	−0.01066 (12)	0.0437 (5)	
H2	0.2910	0.3659	0.0087	0.052*	
C3	0.32285 (14)	0.5010 (2)	−0.03890 (12)	0.0485 (5)	
H3	0.3756	0.4706	−0.0379	0.058*	
N2	0.29823 (12)	0.60467 (17)	−0.06847 (10)	0.0459 (4)	
H2N	0.3334 (18)	0.643 (2)	−0.0811 (15)	0.055*	
C4	0.22400 (13)	0.64928 (19)	−0.07146 (11)	0.0420 (4)	
H4	0.2076	0.7215	−0.0937	0.050*	
C5	0.17111 (12)	0.59364 (17)	−0.04330 (10)	0.0369 (4)	
S1	0.07610 (3)	0.66425 (4)	−0.04649 (2)	0.03510 (15)	
O1	0.08434 (9)	0.69312 (13)	0.02476 (7)	0.0404 (3)	
O2	0.06917 (10)	0.76225 (12)	−0.09190 (8)	0.0426 (3)	
N3	0.00665 (10)	0.56968 (14)	−0.07018 (8)	0.0357 (3)	
C6	−0.00564 (12)	0.51828 (17)	−0.13380 (10)	0.0365 (4)	
O3	0.02024 (10)	0.55425 (13)	−0.18126 (8)	0.0460 (4)	
N4	−0.04911 (12)	0.41930 (16)	−0.14092 (9)	0.0445 (4)	
H4N	−0.0622 (17)	0.389 (2)	−0.1047 (15)	0.053*	
C7	−0.05800 (17)	0.3402 (2)	−0.19833 (12)	0.0533 (6)	
H7	−0.0722	0.3856	−0.2424	0.064*	
C8	−0.1274 (2)	0.2567 (3)	−0.20331 (17)	0.0795 (10)	
H81	−0.1136	0.2106	−0.1607	0.119*	
H82	−0.1346	0.2058	−0.2433	0.119*	
H83	−0.1795	0.2992	−0.2092	0.119*	
C9	0.0240 (2)	0.2749 (3)	−0.1887 (2)	0.0837 (10)	
H91	0.0699	0.3303	−0.1823	0.126*	
H92	0.0186	0.2277	−0.2299	0.126*	
H93	0.0361	0.2251	−0.1477	0.126*	
N1	0.14707 (11)	0.42831 (15)	0.02100 (10)	0.0403 (4)	
H1N	0.0987 (18)	0.452 (2)	0.0161 (14)	0.048*	
C10	0.17350 (13)	0.32972 (18)	0.06475 (11)	0.0404 (4)	
C11	0.12689 (15)	0.23004 (18)	0.04998 (12)	0.0445 (5)	
H11	0.0792	0.2260	0.0095	0.053*	
C12	0.14906 (17)	0.1347 (2)	0.09404 (14)	0.0527 (6)	
C13	0.21970 (18)	0.1432 (2)	0.15223 (14)	0.0602 (7)	
H13	0.2363	0.0787	0.1824	0.072*	
C14	0.26604 (18)	0.2426 (3)	0.16715 (15)	0.0632 (7)	
H14	0.3140	0.2464	0.2074	0.076*	
C15	0.24336 (16)	0.3373 (2)	0.12404 (13)	0.0542 (6)	
H15	0.2750	0.4066	0.1347	0.065*	
C16	0.0971 (2)	0.0273 (2)	0.07844 (19)	0.0770 (9)	
H161	0.1053	−0.0119	0.0384	0.116*	
H162	0.0378	0.0474	0.0679	0.116*	
H163	0.1141	−0.0241	0.1188	0.116*	
C17	0.46551 (12)	0.07450 (16)	0.14989 (10)	0.0361 (4)	
C18	0.45704 (14)	0.05823 (19)	0.21650 (10)	0.0427 (5)	
H18	0.4363	−0.0129	0.2275	0.051*	
C19	0.47838 (14)	0.14364 (19)	0.26474 (11)	0.0437 (5)	
H19	0.4733	0.1307	0.3094	0.052*	
N6	0.50654 (12)	0.24638 (16)	0.25054 (9)	0.0418 (4)	
H6N	0.5204 (16)	0.304 (2)	0.2829 (14)	0.050*	
C20	0.51362 (13)	0.26759 (17)	0.18762 (11)	0.0381 (4)	
H20	0.5332	0.3408	0.1785	0.046*	
C21	0.49306 (12)	0.18543 (16)	0.13642 (10)	0.0344 (4)	
S2	0.50042 (3)	0.21861 (4)	0.05304 (2)	0.03520 (15)	
O5	0.53461 (10)	0.33358 (12)	0.05809 (8)	0.0430 (3)	
O4	0.41666 (9)	0.20622 (13)	0.00468 (8)	0.0438 (3)	
N7	0.55423 (10)	0.11763 (14)	0.03788 (8)	0.0360 (3)	
C22	0.63508 (13)	0.10566 (17)	0.08225 (10)	0.0373 (4)	
O6	0.67497 (9)	0.18090 (13)	0.12360 (7)	0.0432 (3)	
N8	0.66900 (14)	0.00256 (19)	0.07774 (11)	0.0555 (5)	
H8N	0.636 (2)	−0.047 (3)	0.0531 (17)	0.067*	
C23	0.75356 (18)	−0.0308 (3)	0.11811 (16)	0.0696 (8)	
H23	0.7776	0.0268	0.1559	0.084*	
C24	0.7492 (3)	−0.1528 (5)	0.1507 (3)	0.134 (2)	
H241	0.7311	−0.1440	0.1917	0.201*	
H242	0.8046	−0.1890	0.1642	0.201*	
H243	0.7089	−0.2015	0.1164	0.201*	
C25	0.8076 (3)	−0.0405 (5)	0.0741 (3)	0.128 (2)	
H251	0.7865	−0.1020	0.0398	0.192*	
H252	0.8649	−0.0588	0.1031	0.192*	
H253	0.8074	0.0328	0.0500	0.192*	
N5	0.45039 (12)	−0.01004 (14)	0.10282 (9)	0.0404 (4)	
H5N	0.4718 (16)	−0.004 (2)	0.0697 (14)	0.048*	
C26	0.41125 (13)	−0.11707 (16)	0.10831 (10)	0.0370 (4)	
C27	0.45708 (15)	−0.21778 (18)	0.11739 (11)	0.0426 (5)	
H27	0.5151	−0.2154	0.1226	0.051*	
C28	0.41752 (17)	−0.32354 (19)	0.11896 (11)	0.0497 (5)	
C29	0.33272 (17)	−0.3224 (2)	0.11144 (12)	0.0525 (6)	
H29	0.3052	−0.3934	0.1131	0.063*	
C30	0.28749 (16)	−0.2224 (2)	0.10169 (13)	0.0521 (6)	
H30	0.2293	−0.2246	0.0959	0.063*	
C31	0.32645 (14)	−0.11830 (19)	0.10030 (11)	0.0440 (5)	
H31	0.2956	−0.0483	0.0939	0.053*	
C32	0.4664 (2)	−0.4336 (2)	0.12887 (16)	0.0704 (8)	
H321	0.4287	−0.4990	0.1264	0.106*	
H322	0.5100	−0.4328	0.1744	0.106*	
H323	0.4925	−0.4410	0.0923	0.106*	
O8	0.7099 (4)	0.2571 (5)	0.2572 (2)	0.0972 (18)	0.50
H81O	0.740 (6)	0.221 (10)	0.284 (4)	0.146*	0.50
H82O	0.706 (7)	0.228 (10)	0.223 (4)	0.146*	0.50
O7	0.6701 (3)	0.4556 (5)	0.2137 (3)	0.0920 (14)	0.50
H7O	0.679 (6)	0.397 (2)	0.2459 (11)	0.138*	0.50
C33	0.7049 (4)	0.5514 (6)	0.2484 (5)	0.087 (2)	0.50
H331	0.6809	0.5664	0.2856	0.131*	0.50
H332	0.6935	0.6171	0.2164	0.131*	0.50
H333	0.7656	0.5405	0.2685	0.131*	0.50

### Atomic displacement parameters (Å^2^)

**Table:**

	*U*^11^	*U*^22^	*U*^33^	*U*^12^	*U*^13^	*U*^23^
C1	0.0406 (10)	0.0365 (10)	0.0361 (9)	−0.0021 (8)	0.0140 (8)	0.0001 (8)
C2	0.0443 (11)	0.0418 (11)	0.0480 (11)	0.0013 (8)	0.0192 (9)	−0.0009 (9)
C3	0.0451 (11)	0.0534 (13)	0.0518 (12)	−0.0019 (10)	0.0221 (10)	−0.0072 (10)
N2	0.0467 (10)	0.0478 (10)	0.0502 (10)	−0.0092 (8)	0.0254 (8)	−0.0023 (8)
C4	0.0476 (11)	0.0412 (11)	0.0407 (10)	−0.0066 (9)	0.0188 (9)	−0.0011 (8)
C5	0.0421 (10)	0.0360 (10)	0.0341 (9)	−0.0047 (8)	0.0142 (8)	0.0008 (7)
S1	0.0427 (3)	0.0318 (3)	0.0331 (3)	−0.00044 (17)	0.01538 (19)	0.00419 (17)
O1	0.0506 (8)	0.0383 (7)	0.0344 (7)	−0.0024 (6)	0.0165 (6)	0.0000 (6)
O2	0.0553 (8)	0.0328 (7)	0.0412 (8)	−0.0009 (6)	0.0174 (6)	0.0084 (6)
N3	0.0400 (8)	0.0349 (8)	0.0341 (8)	−0.0001 (6)	0.0146 (6)	0.0034 (6)
C6	0.0414 (10)	0.0349 (9)	0.0343 (9)	0.0022 (8)	0.0139 (8)	0.0068 (7)
O3	0.0672 (10)	0.0375 (8)	0.0396 (7)	−0.0043 (7)	0.0258 (7)	0.0047 (6)
N4	0.0593 (11)	0.0420 (10)	0.0351 (9)	−0.0114 (8)	0.0191 (8)	0.0007 (7)
C7	0.0709 (15)	0.0504 (13)	0.0381 (11)	−0.0137 (11)	0.0166 (10)	−0.0022 (9)
C8	0.102 (2)	0.077 (2)	0.0585 (16)	−0.0408 (19)	0.0252 (16)	−0.0156 (15)
C9	0.093 (2)	0.074 (2)	0.081 (2)	0.0160 (17)	0.0221 (18)	−0.0136 (17)
N1	0.0387 (9)	0.0367 (9)	0.0487 (10)	0.0042 (7)	0.0183 (7)	0.0114 (7)
C10	0.0435 (10)	0.0377 (10)	0.0435 (11)	0.0053 (8)	0.0185 (9)	0.0081 (8)
C11	0.0512 (12)	0.0373 (11)	0.0488 (12)	0.0033 (9)	0.0212 (10)	0.0044 (9)
C12	0.0706 (15)	0.0356 (11)	0.0616 (14)	0.0065 (10)	0.0345 (12)	0.0070 (10)
C13	0.0782 (17)	0.0478 (13)	0.0590 (15)	0.0194 (12)	0.0279 (13)	0.0203 (11)
C14	0.0624 (15)	0.0654 (16)	0.0553 (14)	0.0104 (13)	0.0098 (12)	0.0166 (12)
C15	0.0526 (13)	0.0508 (14)	0.0545 (14)	−0.0008 (10)	0.0103 (10)	0.0092 (10)
C16	0.107 (2)	0.0411 (14)	0.089 (2)	−0.0082 (14)	0.0399 (19)	0.0062 (14)
C17	0.0435 (10)	0.0321 (9)	0.0348 (9)	−0.0025 (8)	0.0156 (8)	−0.0036 (7)
C18	0.0576 (12)	0.0384 (10)	0.0365 (10)	−0.0092 (9)	0.0214 (9)	−0.0039 (8)
C19	0.0543 (12)	0.0451 (11)	0.0353 (10)	−0.0058 (9)	0.0192 (9)	−0.0050 (8)
N6	0.0520 (10)	0.0380 (9)	0.0374 (9)	−0.0038 (8)	0.0172 (7)	−0.0111 (7)
C20	0.0434 (10)	0.0323 (9)	0.0404 (10)	−0.0012 (8)	0.0159 (8)	−0.0047 (8)
C21	0.0401 (9)	0.0297 (9)	0.0359 (9)	−0.0021 (7)	0.0159 (8)	−0.0042 (7)
S2	0.0431 (3)	0.0301 (3)	0.0348 (3)	−0.00235 (17)	0.0158 (2)	−0.00089 (16)
O5	0.0552 (8)	0.0292 (7)	0.0493 (8)	−0.0046 (6)	0.0234 (7)	0.0004 (6)
O4	0.0443 (8)	0.0432 (8)	0.0416 (8)	−0.0003 (6)	0.0104 (6)	0.0008 (6)
N7	0.0446 (9)	0.0332 (8)	0.0332 (8)	−0.0022 (7)	0.0166 (7)	−0.0046 (6)
C22	0.0461 (10)	0.0366 (10)	0.0335 (9)	−0.0023 (8)	0.0187 (8)	−0.0019 (7)
O6	0.0512 (8)	0.0379 (7)	0.0385 (7)	−0.0064 (6)	0.0113 (6)	−0.0033 (6)
N8	0.0555 (12)	0.0475 (11)	0.0560 (12)	0.0099 (9)	0.0071 (9)	−0.0164 (9)
C23	0.0596 (15)	0.0695 (18)	0.0684 (17)	0.0205 (13)	0.0045 (13)	−0.0180 (14)
C24	0.101 (3)	0.176 (5)	0.126 (4)	0.047 (3)	0.037 (3)	0.084 (4)
C25	0.087 (3)	0.182 (5)	0.128 (4)	0.060 (3)	0.054 (3)	0.057 (4)
N5	0.0600 (11)	0.0299 (8)	0.0378 (9)	−0.0102 (7)	0.0250 (8)	−0.0059 (7)
C26	0.0514 (11)	0.0313 (9)	0.0307 (9)	−0.0070 (8)	0.0163 (8)	−0.0040 (7)
C27	0.0544 (12)	0.0383 (11)	0.0369 (10)	0.0019 (9)	0.0172 (9)	−0.0022 (8)
C28	0.0794 (16)	0.0329 (11)	0.0373 (11)	0.0006 (10)	0.0192 (10)	−0.0040 (8)
C29	0.0742 (16)	0.0402 (12)	0.0450 (12)	−0.0181 (11)	0.0218 (11)	−0.0050 (9)
C30	0.0544 (13)	0.0528 (14)	0.0514 (13)	−0.0147 (10)	0.0201 (10)	−0.0059 (10)
C31	0.0507 (12)	0.0417 (11)	0.0412 (11)	−0.0013 (9)	0.0168 (9)	−0.0015 (8)
C32	0.103 (2)	0.0414 (13)	0.0661 (17)	0.0128 (14)	0.0255 (16)	0.0007 (12)
O8	0.109 (4)	0.119 (4)	0.042 (2)	0.056 (3)	−0.006 (2)	−0.015 (3)
O7	0.080 (3)	0.090 (3)	0.100 (4)	−0.010 (3)	0.020 (3)	−0.007 (3)
C33	0.049 (3)	0.066 (4)	0.134 (6)	0.007 (3)	0.013 (3)	−0.012 (4)

### Geometric parameters (Å, °)

**Table:**

C1—N1	1.344 (3)	C18—H18	0.9500
C1—C2	1.422 (3)	C19—N6	1.347 (3)
C1—C5	1.428 (3)	C19—H19	0.9500
C2—C3	1.354 (3)	N6—C20	1.345 (3)
C2—H2	0.9500	N6—H6N	0.92 (3)
C3—N2	1.348 (3)	C20—C21	1.371 (3)
C3—H3	0.9500	C20—H20	0.9500
N2—C4	1.337 (3)	C21—S2	1.781 (2)
N2—H2N	0.84 (3)	S2—O5	1.4428 (14)
C4—C5	1.365 (3)	S2—O4	1.4462 (15)
C4—H4	0.9500	S2—N7	1.5704 (16)
C5—S1	1.781 (2)	N7—C22	1.379 (3)
S1—O2	1.4449 (14)	C22—O6	1.248 (2)
S1—O1	1.4491 (14)	C22—N8	1.341 (3)
S1—N3	1.5630 (17)	N8—C23	1.451 (3)
N3—C6	1.378 (3)	N8—H8N	0.85 (3)
C6—O3	1.250 (2)	C23—C25	1.471 (5)
C6—N4	1.344 (3)	C23—C24	1.574 (6)
N4—C7	1.454 (3)	C23—H23	1.0000
N4—H4N	0.90 (3)	C24—H241	0.9800
C7—C8	1.496 (4)	C24—H242	0.9800
C7—C9	1.533 (4)	C24—H243	0.9800
C7—H7	1.0000	C25—H251	0.9800
C8—H81	0.9800	C25—H252	0.9800
C8—H82	0.9800	C25—H253	0.9800
C8—H83	0.9800	N5—C26	1.426 (2)
C9—H91	0.9800	N5—H5N	0.86 (3)
C9—H92	0.9800	C26—C27	1.379 (3)
C9—H93	0.9800	C26—C31	1.385 (3)
N1—C10	1.429 (3)	C27—C28	1.401 (3)
N1—H1N	0.83 (3)	C27—H27	0.9500
C10—C11	1.375 (3)	C28—C29	1.387 (4)
C10—C15	1.392 (3)	C28—C32	1.497 (3)
C11—C12	1.396 (3)	C29—C30	1.367 (4)
C11—H11	0.9500	C29—H29	0.9500
C12—C13	1.387 (4)	C30—C31	1.379 (3)
C12—C16	1.498 (4)	C30—H30	0.9500
C13—C14	1.371 (4)	C31—H31	0.9500
C13—H13	0.9500	C32—H321	0.9800
C14—C15	1.380 (4)	C32—H322	0.9800
C14—H14	0.9500	C32—H323	0.9800
C15—H15	0.9500	O8—H81O	0.75 (5)
C16—H161	0.9800	O8—H82O	0.75 (5)
C16—H162	0.9800	O7—C33	1.346 (8)
C16—H163	0.9800	O7—H7O	0.923 (8)
C17—N5	1.336 (2)	C33—H331	0.9800
C17—C18	1.419 (3)	C33—H332	0.9800
C17—C21	1.423 (3)	C33—H333	0.9800
C18—C19	1.359 (3)		
			
N1—C1—C2	121.69 (19)	C19—C18—H18	119.7
N1—C1—C5	122.07 (18)	C17—C18—H18	119.7
C2—C1—C5	116.25 (18)	N6—C19—C18	121.33 (19)
C3—C2—C1	120.7 (2)	N6—C19—H19	119.3
C3—C2—H2	119.7	C18—C19—H19	119.3
C1—C2—H2	119.7	C20—N6—C19	120.66 (18)
N2—C3—C2	120.9 (2)	C20—N6—H6N	117.9 (17)
N2—C3—H3	119.6	C19—N6—H6N	121.4 (17)
C2—C3—H3	119.6	N6—C20—C21	121.13 (19)
C4—N2—C3	121.00 (19)	N6—C20—H20	119.4
C4—N2—H2N	121 (2)	C21—C20—H20	119.4
C3—N2—H2N	117.2 (19)	C20—C21—C17	120.05 (18)
N2—C4—C5	121.6 (2)	C20—C21—S2	119.65 (15)
N2—C4—H4	119.2	C17—C21—S2	120.30 (14)
C5—C4—H4	119.2	O5—S2—O4	114.89 (9)
C4—C5—C1	119.49 (19)	O5—S2—N7	117.42 (9)
C4—C5—S1	117.85 (16)	O4—S2—N7	106.96 (9)
C1—C5—S1	122.59 (15)	O5—S2—C21	106.24 (9)
O2—S1—O1	114.78 (9)	O4—S2—C21	106.00 (9)
O2—S1—N3	117.08 (9)	N7—S2—C21	104.24 (9)
O1—S1—N3	107.04 (9)	C22—N7—S2	117.27 (13)
O2—S1—C5	105.84 (9)	O6—C22—N8	121.3 (2)
O1—S1—C5	105.79 (9)	O6—C22—N7	125.21 (18)
N3—S1—C5	105.32 (9)	N8—C22—N7	113.48 (18)
C6—N3—S1	117.75 (14)	C22—N8—C23	124.2 (2)
O3—C6—N4	120.94 (19)	C22—N8—H8N	116 (2)
O3—C6—N3	126.50 (19)	C23—N8—H8N	119 (2)
N4—C6—N3	112.55 (17)	N8—C23—C25	111.3 (3)
C6—N4—C7	122.53 (18)	N8—C23—C24	107.9 (3)
C6—N4—H4N	120.4 (18)	C25—C23—C24	108.0 (4)
C7—N4—H4N	115.1 (18)	N8—C23—H23	109.9
N4—C7—C8	109.9 (2)	C25—C23—H23	109.9
N4—C7—C9	110.6 (2)	C24—C23—H23	109.9
C8—C7—C9	110.0 (3)	C23—C24—H241	109.5
N4—C7—H7	108.8	C23—C24—H242	109.5
C8—C7—H7	108.8	H241—C24—H242	109.5
C9—C7—H7	108.8	C23—C24—H243	109.5
C7—C8—H81	109.5	H241—C24—H243	109.5
C7—C8—H82	109.5	H242—C24—H243	109.5
H81—C8—H82	109.5	C23—C25—H251	109.5
C7—C8—H83	109.5	C23—C25—H252	109.5
H81—C8—H83	109.5	H251—C25—H252	109.5
H82—C8—H83	109.5	C23—C25—H253	109.5
C7—C9—H91	109.5	H251—C25—H253	109.5
C7—C9—H92	109.5	H252—C25—H253	109.5
H91—C9—H92	109.5	C17—N5—C26	124.70 (17)
C7—C9—H93	109.5	C17—N5—H5N	117.8 (18)
H91—C9—H93	109.5	C26—N5—H5N	116.9 (18)
H92—C9—H93	109.5	C27—C26—C31	121.27 (19)
C1—N1—C10	124.46 (18)	C27—C26—N5	119.60 (19)
C1—N1—H1N	119.7 (19)	C31—C26—N5	119.01 (19)
C10—N1—H1N	115.8 (19)	C26—C27—C28	119.6 (2)
C11—C10—C15	120.5 (2)	C26—C27—H27	120.2
C11—C10—N1	119.59 (19)	C28—C27—H27	120.2
C15—C10—N1	119.82 (19)	C29—C28—C27	118.0 (2)
C10—C11—C12	120.5 (2)	C29—C28—C32	121.7 (2)
C10—C11—H11	119.7	C27—C28—C32	120.3 (2)
C12—C11—H11	119.7	C30—C29—C28	122.0 (2)
C13—C12—C11	118.2 (2)	C30—C29—H29	119.0
C13—C12—C16	121.5 (2)	C28—C29—H29	119.0
C11—C12—C16	120.3 (3)	C29—C30—C31	119.9 (2)
C14—C13—C12	121.4 (2)	C29—C30—H30	120.0
C14—C13—H13	119.3	C31—C30—H30	120.0
C12—C13—H13	119.3	C30—C31—C26	119.1 (2)
C13—C14—C15	120.4 (3)	C30—C31—H31	120.4
C13—C14—H14	119.8	C26—C31—H31	120.4
C15—C14—H14	119.8	C28—C32—H321	109.5
C14—C15—C10	119.1 (2)	C28—C32—H322	109.5
C14—C15—H15	120.5	H321—C32—H322	109.5
C10—C15—H15	120.5	C28—C32—H323	109.5
C12—C16—H161	109.5	H321—C32—H323	109.5
C12—C16—H162	109.5	H322—C32—H323	109.5
H161—C16—H162	109.5	H81O—O8—H82O	105.0 (10)
C12—C16—H163	109.5	C33—O7—H7O	107.0 (7)
H161—C16—H163	109.5	O7—C33—H331	109.5
H162—C16—H163	109.5	O7—C33—H332	109.5
N5—C17—C18	122.11 (18)	H331—C33—H332	109.5
N5—C17—C21	121.63 (17)	O7—C33—H333	109.5
C18—C17—C21	116.25 (17)	H331—C33—H333	109.5
C19—C18—C17	120.50 (19)	H332—C33—H333	109.5
			
N1—C1—C2—C3	−176.8 (2)	N5—C17—C18—C19	175.5 (2)
C5—C1—C2—C3	3.5 (3)	C21—C17—C18—C19	−3.0 (3)
C1—C2—C3—N2	−1.9 (3)	C17—C18—C19—N6	1.2 (4)
C2—C3—N2—C4	−0.8 (3)	C18—C19—N6—C20	0.6 (3)
C3—N2—C4—C5	1.7 (3)	C19—N6—C20—C21	−0.5 (3)
N2—C4—C5—C1	0.1 (3)	N6—C20—C21—C17	−1.5 (3)
N2—C4—C5—S1	176.95 (16)	N6—C20—C21—S2	178.14 (16)
N1—C1—C5—C4	177.7 (2)	N5—C17—C21—C20	−175.4 (2)
C2—C1—C5—C4	−2.6 (3)	C18—C17—C21—C20	3.1 (3)
N1—C1—C5—S1	1.0 (3)	N5—C17—C21—S2	5.0 (3)
C2—C1—C5—S1	−179.31 (15)	C18—C17—C21—S2	−176.49 (16)
C4—C5—S1—O2	10.41 (19)	C20—C21—S2—O5	3.78 (19)
C1—C5—S1—O2	−172.81 (16)	C17—C21—S2—O5	−176.60 (16)
C4—C5—S1—O1	−111.79 (17)	C20—C21—S2—O4	−118.87 (17)
C1—C5—S1—O1	64.99 (18)	C17—C21—S2—O4	60.75 (18)
C4—C5—S1—N3	135.05 (17)	C20—C21—S2—N7	128.44 (17)
C1—C5—S1—N3	−48.17 (18)	C17—C21—S2—N7	−51.94 (18)
O2—S1—N3—C6	57.06 (17)	O5—S2—N7—C22	54.98 (17)
O1—S1—N3—C6	−172.47 (14)	O4—S2—N7—C22	−174.19 (14)
C5—S1—N3—C6	−60.20 (16)	C21—S2—N7—C22	−62.19 (16)
S1—N3—C6—O3	−15.8 (3)	S2—N7—C22—O6	−16.3 (3)
S1—N3—C6—N4	163.11 (15)	S2—N7—C22—N8	163.34 (16)
O3—C6—N4—C7	9.4 (3)	O6—C22—N8—C23	−1.3 (4)
N3—C6—N4—C7	−169.6 (2)	N7—C22—N8—C23	179.1 (2)
C6—N4—C7—C8	−164.5 (2)	C22—N8—C23—C25	−110.2 (4)
C6—N4—C7—C9	73.9 (3)	C22—N8—C23—C24	131.5 (3)
C2—C1—N1—C10	10.9 (3)	C18—C17—N5—C26	11.2 (3)
C5—C1—N1—C10	−169.43 (19)	C21—C17—N5—C26	−170.33 (19)
C1—N1—C10—C11	−125.7 (2)	C17—N5—C26—C27	−112.5 (2)
C1—N1—C10—C15	57.9 (3)	C17—N5—C26—C31	71.3 (3)
C15—C10—C11—C12	−0.2 (3)	C31—C26—C27—C28	−0.4 (3)
N1—C10—C11—C12	−176.6 (2)	N5—C26—C27—C28	−176.50 (18)
C10—C11—C12—C13	−0.9 (3)	C26—C27—C28—C29	−0.2 (3)
C10—C11—C12—C16	178.8 (2)	C26—C27—C28—C32	−179.9 (2)
C11—C12—C13—C14	1.1 (4)	C27—C28—C29—C30	1.0 (3)
C16—C12—C13—C14	−178.5 (3)	C32—C28—C29—C30	−179.3 (2)
C12—C13—C14—C15	−0.3 (4)	C28—C29—C30—C31	−1.1 (4)
C13—C14—C15—C10	−0.9 (4)	C29—C30—C31—C26	0.5 (3)
C11—C10—C15—C14	1.1 (4)	C27—C26—C31—C30	0.2 (3)
N1—C10—C15—C14	177.5 (2)	N5—C26—C31—C30	176.38 (19)

### Hydrogen-bond geometry (Å, °)

**Table:**

*D*—H···*A*	*D*—H	H···*A*	*D*···*A*	*D*—H···*A*
N1—H1N···N3	0.83 (3)	2.37 (3)	2.986 (2)	131 (2)
N1—H1N···N3^i^	0.83 (3)	2.38 (3)	3.060 (2)	139 (2)
N2—H2N···O5^ii^	0.84 (3)	2.14 (3)	2.847 (2)	142 (3)
N2—H2N···O6^ii^	0.84 (3)	2.21 (3)	2.822 (2)	130 (2)
N4—H4N···O1^i^	0.90 (3)	2.02 (3)	2.919 (2)	174 (3)
N5—H5N···N7	0.86 (3)	2.21 (3)	2.910 (2)	138 (2)
N5—H5N···N7^iii^	0.86 (3)	2.47 (3)	3.098 (2)	130 (2)
N6—H6N···O2^iv^	0.92 (3)	2.53 (3)	3.036 (2)	115 (2)
N6—H6N···O3^iv^	0.92 (3)	1.79 (3)	2.667 (2)	158 (2)
N8—H8N···O4^iii^	0.85 (3)	2.21 (3)	3.034 (3)	164 (3)
O7—H7O···O8	0.92 (1)	1.70 (5)	2.480 (8)	140 (7)
O7—H7O···O8^v^	0.92 (1)	2.50 (10)	2.995 (9)	114 (8)
O8—H82O···O6	0.75 (5)	2.00 (6)	2.735 (5)	166 (11)
O8—H81O···O6^v^	0.75 (5)	2.02 (6)	2.721 (5)	158 (12)

Symmetry codes: (i) −*x*, −*y*+1, −*z*; (ii) −*x*+1, −*y*+1, −*z*; (iii) −*x*+1, −*y*, −*z*; (iv) *x*+1/2, −*y*+1, *z*+1/2; (v) −*x*+3/2, *y*, −*z*+1/2.
